# Do immigrants know less than natives about cancer screening tests? – the case of Netherlands

**DOI:** 10.1016/j.jmh.2024.100258

**Published:** 2024-07-29

**Authors:** Dr Jelena Arsenijevic, Dr Verena Seibel

**Affiliations:** a Faculty of Law, Economics and Governance*,* Utrecht University*,* Utrecht*,* the Netherlands; b Faculty of Social and Behavioral Science, Utrecht University*,* the Netherlands

**Keywords:** Migrants, Screening test, Cancer, Equity

## Abstract

**Introduction:**

The Netherlands was one of the first countries in Europe to offer breast, colorectal and cervical cancer screening tests free of charge. Yet, a significant share of migrants in the Netherlands forgo the use of these preventive screenings. Qualitative research suggests, that lack of system knowledge on how the healthcare system operates (e.g. age eligibility of cancer screenings), is one factor contributing to this underuse among migrants. However, little is known about the extent to which migrants differ from natives in their system knowledge and about potential causes of this ethnic gap. The contribution of this study is therefore twofold: First, we examine whether migrants in the Netherlands have lower system knowledge regarding cancer screenings than the natives. Second, we examine which factors explain potential ethnic differences in system knowledge between migrants and non-migrants.

**Method:**

Using the Longitudinal Internet Study for Social Sciences (LISS), we matched newly collected data on system knowledge about healthcare with Health module (wave 14). To assess the difference in system knowledge among migrants and non-migrants, while taking into account potential selection bias, we applied propensity score matching, one-to-one matching procedure with no replacement. We compared first generation non - Western migrants (FNWM) and second-generation non-western migrants (SNWM) with their most similar non-migrants group regarding their knowledge about breast, colorectal, and cervical screening. A Blinder-Oaxaca decomposition with non-matched samples was used to examine which factors can explain the differences in system knowledge among migrants and non-migrants.

**Results:**

Our results show that first generation migrants have lower system knowledge about all three screening tests, while second generation migrants differ from non-migrants only with regard to knowledge about breast and colorectal cancer screenings. The ethnic differences found are not caused by personal or social factors such as education or income.

**Conclusion:**

We recommend Dutch healthcare policy makes to focus in the strengthen of cancer screening system knowledge among migrant populations in order to increase their participation in these screening programs.

## Highlights

• Migrants and non-migrants differ significantly in their system knowledge about cancer preventive screening programs.
• The difference is observed in case of breast cancer screening tests and colorectal cancer screening tests.
• Second generation non-Western migrants do not differ in system knowledge about cervix cancer screening test from non-migrants.
• Current dutch policy is mostly focus on cancer awareness and knowledge about test procedure, but does not capture some structural aspects such as information about system organization, dissemination of relevant and factual knowledge through educational system or informal channels.

## Introduction

Cancer is one of the leading causes of death in developed countries. In 2019, 7,2 millions of new cases of cancer were registered in OECD countries, while the numbers estimated by WHO suggested 20 million new cancer cases in the world in 2022 with 35 million new cases predicted for 2050 (Ferlay et al., 2015; Sung et al., 2021; WHO, 2024). Common cancer deaths are related to lung cancer – 21.5 %, breast cancer – 14.5 %, colorectal cancer 11 %, while mortality rate is much lower for cervical cancer 1.9 % (Indicators, 2021). The reasons for lower mortality rate for cervical cancer are related to new therapeutic approaches, vaccination and early diagnosis via screening. With vaccination against human papilloma virous for young girls and regular screening for adult women, cervical cancer is today perceived by many clinicians as a chronic and treatable disease, rather than cause of death (Buskwofie et al., 2020; Vu et al., 2018). A similar situation is observed for breast and colorectal cancer. Particularly prevention screening tests allow both patients and clinicians to identify cancer in early stages, which increases chances for successful treatment and survival. In many OECD countries, screening tests are organized as population prevention programs and are usually distributed free of charge (Gmeinder et al., 2017). Although these positive effects of screening tests are widely recognized, previous literature has shown that certain vulnerable population groups do not adhere to screening tests. Migrants who live in developed countries are significantly less likely to make use of these screening tests than natives (Adunlin et al., 2019; Buskwofie et al., 2020; Ferdous et al., 2018; Gmeinder et al., 2017; Gong et al., 2022; Hertzum-Larsen et al., 2019; Van Hemelrijck et al., 2019). This means that cancer among migrants is usually discovered in the late stages when treatment possibilities are limited.

The underuse of cancer preventive screening programs among migrant populations might also explain the ‘cancer paradox’ among the migrant population. On the one hand, migrants are found to have lower incidence rates for breast, lung, colorectal, and cervical cancer than natives (Norredam et al., 2014). This finding is in accordance with theory of the “healthy migrant effect” – people who decided to emigrate are usually younger and healthier than those who stay in their country of origin and also than the native population, who mirrors a very diversified demographic composition of all ages and health status (Kennedy et al., 2006). On the other hand, the mortality rate due to cancer is higher for migrants in developed countries than for non-migrants (Indicators, 2021). One prominent explanation for this cancer paradox lies in the fact that migrants make much less use of preventive cancer screening programs than natives, thereby reducing early detection and proper treatment (Marques et al., 2020). This situation has inspired many practitioners and researchers to examine why migrants do not attend these screenings. This has resulted in a huge number of literature that have identified mostly personal characteristics of individuals (insecurity, fear of the procedure), behavioral characteristics (lack of pro-active health behavior), gain-pain trade-off – as some procedures are painful, and cultural and religious reasons (Hartman et al., 2009).

However, those studies did not examine how much migrants know about the healthcare system and potential possibilities to access cancer preventive screening programs. Ethnic variation in so-called *system knowledge*- knowledge about the organization of a welfare service, the eligibility criteria, and the access possibilities knowledge (Seibel, 2019; Seibel and Renema, 2021; Seibel, 2021, 2023) potentially contributes to ethnic inequality within the healthcare. A possible lack of system knowledge about healthcare can have detrimental consequences for migrant populations, particularly in the case of cancer prevention screenings: If, for example, migrants do not understand when they are eligible for cancer screening programs, their likelihood of participating most likely decreases (Berens et al., 2016; Hamdiui et al., 2022; Woudstra et al., 2016).

Although lack of system knowledge about preventive screenings is recognized as one of the barriers to forgo screening tests, the current literature poses two research gaps: First, most research referring to migrants’ lack of knowledge is of qualitative nature and refers to very specific migrant groups (Ferdous et al., 2018; Hamdiui et al., 2022; marques et al., 2020). Hence, we do not know if lack of system knowledge is a generalizable finding across several migrant groups who potentially differ in healthcare culture and behavior. Second, we do not know what contributes to the ethnic gap in system knowledge. Some authors argue that possible reasons are lower education levels and lower health literacy among migrants (Mehrara et al., 2022; Willis et al., 2016). Others argue that the general use of informal online sources to find relevant health information may be a reason for lower knowledge (Arsenijevic et al., 2020). To our understanding, due to the lack of data, a systematic analysis of system knowledge about preventive cancer screening programs among migrant populations in comparison to non-migrants is missing.

The aim of this study is therefore two-fold: First, we aim to examine to what extent migrants differ in their system knowledge about screening procedures for breast, colorectal and cervical cancer from their native non-migrant counterparts in the Netherlands. We thereby focus on the ‘eligibility’-aspect of system knowledge, by examining knowledge about the main eligibility criteria for cancer screening procedures, namely age. We thereby examine migrants with non-Western background who are found to be the most vulnerable group in the Dutch healthcare system (lower access to healthcare services, lower health status, lower life expectancy) (Ruijer and Arsenijevic, 2020). Second, we explore which factors contribute to potential ethnic differences in system knowledge. The healthy migrant effect discussed above shows that people who decide to emigrate are not randomly selected in their country of origin but biased with regards to better health. As a result, a simple comparison between migrants with non-migrants regarding their knowledge about screening procedures can lead to selection bias. Previous studies that have examined migrants’ system knowledge about screening procedures for cervical, breast and colorectal cancer in their host countries have relied predominately on qualitative studies, which, despite their richness, do not control for this selection biases (Borjas et al., 2019; Woudstra et al., 2016). In this study, we are bridging this gap by analyzing unique quantitative data collected via the Longitudinal Internet Study for Social Sciences (LISS) on migrants’ and non-migrants system knowledge living in the Netherlands with a probability-based sample. We apply a propensity score matching procedure that will allow us to compare migrants with non-migrants of similar age, educational level, gender and income. In this way we are able to control for selection biases that are usually overlooked (Borjas et al., 2019). Furthermore, we will examine potential factors that are related to migrants knowledge about screening procedures, such as how migrants acquire healthcare related information, their level of education and health literacy. Last, but not least, we examine to what extent these factors explain potential ethnic differences in system knowledge. For this purpose we will use logistic regression on matched sample and Blinder - Oaxaca decomposition index on non-matched samples (Sinning et al., 2008).

## Background

In a broader welfare-related context, system knowledge refers to people's understanding of the organisation of a welfare service, its eligibility criteria and access possibilities (Seibel, 2019; Seibel et Renema, 2020; Seibel, 2021; Seibel, 2023). Assessment of system knowledge in healthcare is a relatively new and bounded by complexity of healthcare systems all over the world (Seibel, 2019; Seibel & Renema,2020; Willis et al., 2016). This means that system knowledge can encompass different aspects of the healthcare system such as understandings about access rights, financial coverage, or procedures (Seibel, 2019; Willis et al., 2016). With regards to the complexity of the healthcare system and the diversity between different healthcare systems, it is not surprising that vulnerable population groups such as migrants tend to lack relevant information in terms of system knowledge (Bell et al., 2019). System knowledge is thereby to be distinguished from other types of knowledge concepts such as administrative knowledge referring to the knowledge welfare providers possess about the healthcare regulations (see, for example, Abeje and Azage 2015) or patients' knowledge about health in general and treatments (Wake 2020).

The complex nature of system knowledge and healthcare systems have also influenced the design of the previous research. Most researchers opted for a qualitative research design in order to capture the complex nature of knowledge (Mehrara et al., 2022; Patel et al., 2023; Straiton and Myhre, 2017; Xiang et al., 2023). Besides the qualitative research design, previous studies have focused on healthcare services related to curative care such as services from general practitioners or maternity care (Harding et al., 2017; Mehrara et al., 2022). The examination of system knowledge within preventive healthcare services seems to be rare and also bounded with additional challenges (Bell et al., 2019). Prior studies mainly focused on health behavior, understanding factors leading to lower attendance among migrants in cancer screening, identifying insufficient system knowledge as one of the key contributors (Berens et al., 2016; Hartman et al., 2009; Marques et al., 2020, 2020; Mehrara et al., 2022; Özkan and Taylan, 2021; Straiton and Myhre, 2017; Woudstra et al., 2016).

Since these studies focused on explaining migrants’ behavior, they relied on concepts that derived from health promotion theories. This implies that most of the previous research that reported some aspects of system knowledge was predominately focused on health promotion theories such as the Health Beliefs Models (perceived benefits, perceived barriers), Theory of Planned behaviors (attitudes, perceived behavioral control), Socio-ecological models (system knowledge as a part of knowledge about the environment) or Social Cognitive theories (system knowledge as a part of norms and awareness)(Dsouza et al., 2022). According to the most often used Theory of Health Belief Model (THBM) (Rosenstock et al., 1988), knowledge about illness and knowledge about treatment possibilities and their benefits are a crucial prerequisite for people following medical advice, such as participating in cancer preventive screening programs. Previous literature finds that indeed, migrants’ lack of knowledge about screening procedures is linked to lower participation rates in cancer preventive screening programs (Berens et al., 2016; Hartman et al., 2009; Marques et al., 2020; Özkan and Taylan, 2021). Using focus groups, Hamidiu et al. (2022) identify migrants' knowledge barriers about cervical cancer screenings, including knowledge about who is performing screening tests, whether mammography is performed by both male and female professionals, and whether the procedure is harmful (Hamdiui et al., 2022). Recent literature also shows that procedural knowledge about screenings increases people's awareness of cancer and the related risks (Özkan and Taylan, 2021). Another study based on qualitative data examined migrants’ knowledge about colorectal cancer screening procedures and measure knowledge about diseases through the concept of perceived severity (Austin et al., 2009). Woudstra et al. (2016) also find that knowledge about colorectal cancer as a severe disease is seen as a precursor for migrants to participate in screening tests. Another study conducted in Germany examined reasons for non-attendance to mammography among Turkish immigrant women. They reported a lack of knowledge about organized mammography screening as one of the main causes of non-attendance (Berens et al., 2016). In many cases similar to the one mentioned above, the focus is on knowledge about the disease and knowledge about the screening test procedure, where the latter one includes some aspects of system knowledge (Salad et al., 2015).

These mainly qualitative studies have opened an important window by emphasizing the relevance of system knowledge among migrants and suggesting that system knowledge should be measured as a separate and independent concept (Hamdiui et al., 2022; Özkan and Taylan, 2021). However, until now we lack a systematic comparison between migrant and native populations on system knowledge. In addition, explanations for this ethnic gap in system knowledge remain unclear. Seibel (2019) finds that particularly lower levels of education and low language skills are responsible for migrants’ lack of knowledge about their social right to access healthcare. However, research about system knowledge from other welfare domains, such as childcare (Seibel, 2021) shows that social contacts are also crucial for acquiring system knowledge and are likely to play an important role in transferring knowledge about cancer prevention screening programs. However, these studies do not refer to cancer preventive screening programs and thus lack potentially relevant explanatory factors such as channels through which healthcare information is acquired. To conclude, while previous studies emphasize a number of factors that potentially contribute to lower system knowledge among migrants, these factors are not explored yet in the context of cancer preventive screening programs (Suphanchaimat et al., 2015). Our second contribution lies therefore in the testing of various explanatory factors for the potential ethnic gap in cancer-related system knowledge.

## System knowledge about preventive screening tests among migrants – the Dutch context

Public healthcare services in the Netherlands are the responsibility of the national and local government. According to The Public Health Law (“Wet publieke gezondheid”) (wpg) enacted in 2008, the major responsibility is given to local municipalities and their Municipal Public Health Service -GGD (Gemeentelijke gezondheidsdienst) (Arsenijevic and Groot,2017). Preventive services such as the national vaccination program or cancer screening tests are financed through general taxes, while some selective prevention services are financed through the insurance system. Since 2006, the Netherlands switched from social insurance system towards a system of managed competition where goverments has a regulatory role. All citizens are obliged to buy a basic insurance package from one out of nine insurance companies. The government plays the role of the regulator and, together with scientific institutions such as the Institute for Public Health (RIVM), decides which selective prevention services are included in the basic insurance package. Preventive screening tests for breast, colorectal and cervical cancer are part of population programs, being organized by the Ministry of Health and implemented via the Institute for Public Health (RIVM). This means that they are financed through general taxes and they are free of charge for users.

The breast cancer screening test was first introduced in the Netherlands in 1988, when the Dutch public health institute started with the promotion of the national population program and when the first invitation letter was sent to eligible women (Fracheboud et al., 2001). The national screening program for cervical cancer was introduced in 1996 as a Papanicolaou test. The Netherlands was one among the first countries in EU to introduce HPV screening in 2017 (Hamdiui et al., 2022). Women receive their first invitation for a cervical cancer screening from the age of 30 and from this point will be invited every 5 years. The colorectal cancer screening was introduced gradually since 2014 and it was among the last introduced preventive screenings (Bronzwaer et al., 2019). Both men and women age 55 – 70 are eligible for colorectal cancer screening once in two years. For all three preventive tests, the beginning of the procedure is the same – people eligible by their age are invited to the specific screenings via a letter that is delivered to their home address. If the person does not respond to the letter, a new reminder is sent within six weeks. Every individual that is invited for a screening test has to be registered in the Personal records database (Basisregistratie Personen; https://www.rijksoverheid.nl/onderwerpen/privacy-en-persoonsgegevens/basisregistratie-personen-brp). Also, all eligible people can subscribe or unsubscribe from the screening procedure by contacting the national screening organization via phone, email or through the SceenIT programme (https://www.rivm.nl/en/population-screening-programmes/source-code-released).

However, despite the various efforts to inform people about their eligibility to participate in these cancer preventive screening programs, this information might not always translate into knowledge, particularly among migrant populations. Migrants are more likely to move internationally and within the country of residence than non-migrants, leading to a change of addresses and very often a reduced trace in the personal records database (de Hoon et al., 2021; de Jong and de Valk, 2023). If the new address is not communicated on time to the municipality and the new general practitioner (GP), invitations to the cancer preventive screenings do not reach their target group. Furthermore, knowledge about screening eligibility is needed in order to know when to contact the GP and/or national screening organization for an invitation letter. For some groups, this can lead to a vicious circle of knowledge gaps which can negatively influence participation in cancer prevention screening programs.

## Data

In this paper we will use the data from the Longitudinal Internet Studies for Social Sciences (LISS) panel study. The LISS panel represents a robust dataset comprising of 5000 households, encompassing approximately 7500 individuals who are permanent residents of the Netherlands and primarily communicate in the Dutch language. Rigorous sampling techniques were employed in collaboration with the Dutch Central Bureau of Statistics (CBS) to randomly select participants from the population register. To ensure comprehensive representation, all individuals within the sample were cordially invited to participate via a written invitation letter, followed by a personalized contact through telephone or in-person visits, depending on the available means within each household. Notably, households lacking access to the internet or computer resources were not excluded from participation, as they were provided with a computer device known as simPC.

Every month, panel members are invited to participate in online questionnaires, distributed by the LISS panel. Respondents are compensated for their participation and efforts, receiving payment for each completed survey. Furthermore, proactive measures are implemented to establish contact with panel members who have exhibited limited activity or engagement within the panel, ensuring their continued involvement in the panel. More information about the data collection, sample procedure, and representativeness can be found on the webpage Lissdata and within other relevant literature (https://www.lissdata.nl/about-panel(Scherpenzeel, 2011)

In fall 2022, we collected data on healthcare system knowledge with the LISS panel (‘Barriers to healthcare’ module), thereby contributing to the already existing health modules on mental health, physical health, lifestyle behavior, preventive care, and so forth. For the purpose of this study, we have developed a new questionnaire capturing among others respondents’ knowledge about the age eligibility criteria for participation in cancer preventive screening programs. The detailed description of this questionnaire can be found in Supplementary file.

The ‘Barriers to healthcare’ module consists of a sub sample of *n* = 614 respondents. Among this 614 individuals, we distinguish three groups of respondents: a) people without a migration background, who have been born in the Netherlands (*n* = 312), b) first generation migrants who have been born in another non-Western country with residence in the Netherlands (*n* = 160) and c) second-generation migrants who have been born in the Netherlands, but of whom at least one of the parents was born in another non-Western country (*n* = 142) (CBS 2022). After list-wise deletion, we analyzed a final sample of 610 respondents.

## Measurements

To examine whether migrants and non-migrants in the Netherland differ in their knowledge about cancer screenings, respondents were asked to answer the following question: “As part of the so-called national population screening programme, RIVM invites everyone living in the Netherlands to take part in various preventive medical examinations to detect cancer at an early stage so that prompt treatment can be provided. What do you think, at what age do the following groups get their first invitation to such a population screening? 1. Women for breast cancer screening, 2. Men and women for colorectal cancer screening and 3. Women for cervical cancer screening.” For each of the three dependent variables, multiple answers were given as examples for the correct eligible age (see Supplementary file). We have recorded each dependent variable in dummies – where the correct answer is coded as 1 and all non-corrected answers are coded as 0. For example, for breast cancer screening answer “since women become 50 old, she is eligible for first mammography” is coded as 1, while all other answers are coded as 0.

We are aware that those 3 variables do not capture the complex concept of the eligibility-aspect of system knowledge – for example we did not ask participants about their knowledge about other formal requirements of cancer preventive screening participation, such as registration with the municipality. However the previous studies have shown that one-item knowledge question with a clear answer (age number, for example) are equally good predictors as multi-item scales (Gardner et al., 1998). We thus chose the eligibility criteria which is most relevant for cancer preventive screening programs, namely age.

We first compare differences in knowledge about breast cancer screening, cervical cancer screening and colorectal cancer screening between first-generation non-Western migrants and non-migrants using propensity score matching (PSM). We repeat the same analysis to compare the differences in knowledge between second-generation non-Western migrants and non-migrants, thus creating two subsamples.

Previous literature has shown that PSM is more robust and precise than standard logistic regression (Borjas et al.,2019). In this article, we use PSM to address a potential selection bias. Selection bias arises because migrants are not randomly selected from the population in their country of origin. People who migrate are on average younger and healthier than people who decide to stay in their country of origin. Similarly, this means that migrants tend to differ in observable characteristics such as gender, age, income or education (so called socio-demographic characteristics) from randomly chosen non-migrants. As a result, they cannot be compared with a randomly selected non-migrant person based on their preventive knowledge. The selection-bias that arises from observable characteristics can influence migrant status (our independent variable) but can also influence migrants’ system knowledge about screenings (dependent variable) (for example higher educated migrants have better knowledge than lower educated non-migrants). This is known as the confounder effect. Propensity score matching attempts to account for selection bias that is related to both independent and dependent variables. We have assured that by applying propensity score matching we compare migrants with the most similar group of non-migrants. In this way, we can assume that the difference in system knowledge towards prevention screenings is related to migrant status, rather than observable socio-demographic characteristics.

PSM consists of several steps – first we created dummy variables to identify the relevant treatment and control groups. We created the variable t1, where the group of non-migrants is a control group (coded as 0) and first-generation non-Western migrants is a treatment group (coded as 1). Then, we created the variable t2 where the group of non-migrants is a control group (coded as 0) and second-generation non-Western migrants as the treatment group (coded as 1). These identification variables are used to create two subsamples: first-generation migrants versus non-migrants and second-generation migrants vs non-migrants. Then, based on previous literature, we have identified relevant covariates for matching – for both treatment groups we used gender, age, income, and level of education as matching covariates (Marques et al., 2020). These variables are taken from the general LISS panel dataset and they have been merged with our Barriers to Healthcare module. After that, we have calculated the propensity score (PS). In our case, the propensity score is a predicted probability using a regression on migrant/non-migrant status. To estimate the PS we use probit regression applying STATA command psmatch2. We run this probit regression six times (for each outcome) for both subsamples. Based on propensity score (PS) (using 1:1 matching without replacement) we have matched similar individuals from treatment and control groups and estimated the treatment effects on outcome (each of the three knowledge variables), comparing the matched groups. Additionally, to assess whether PSM is addressing adequately the problem of selection bias, we have performed balancing tests using STATA command pstest. This test shows whether biases imposed by covariates are reduced after the matching. We present the results from balancing tests for all three knowledge variables in Supplementary file.

After matching first-generation non- Western migrants with the most similar non-migrants, we have calculated the difference in knowledge about breast, colorectal and cervical cancer screening between them. We have repeated the same procedure to compare the difference in cancer screening knowledge between second generation non-Western migrants and non-migrants. For that purpose we estimated the average treatment effect (ATT) for all three dependent variables using STATA command teffects. In case where all characteristics of all individuals are equally distributed between the migrants and non-migrants (SB ≈ 0), the ATT can be calculated as the average difference in outcome (in our case, knowledge about the eligible age for cancer screening) between the migrants and non-migrants:

$$\text{ATT}=\;\frac{1}{N}\sum_{i=1}^{N}\left(Yi1-Yio\right)$$

where Y_i1_ represents the average outcome of migrants, Y_i0_ the average outcome of non-migrants, and N the number of matched individuals. For each sub-sample we present the ATT results.

In the next step we ran a logistic regression on the matched samples for all three dependent variables. As covariates, we have used three groups of variables that were available from our Barriers to Healthcare module: 1. previous experience with screenings - whether the person has already had a preventive screening for breast, cervical or colorectal cancer, 2. use of informal network for getting relevant information about the healthcare system and procedures - asking friends, family members or colleagues and neighbours about healthcare system and procedures and 3. use of formal sources for getting relevant information about healthcare systems and procedures – where the respondents look for information about healthcare system and procedures – on websites from their insurance company, on web platforms of public health centres (GGD) or on the health specialized web platform known as zorgkaart. As a sensitivity check we also present results for standard logistic regression for unmatched samples (see Supplementary file).

Descriptive statistics for all variables used in propensity score analysis is presented in Table 1.

**Table 1:** Descriptive statistics for all variables included in the study.

Variable name	Mean/median (SD)
Age	47.25 (14.68)
man	0.51
women	0.49
Level of education in CBS categories *Basic school to 6-university education*	3.98 (1.5)
Neto income per month	1771.27 (1277.12)
*How often do you search for information about healthcare system via your friends (v8) *1-very unlikely to 4-very likely;*	2.68 (1.04)
*How often do you search for information about healthcare system via your family members? (v9) *1-very unlikely to 4-very likely;*	3.04 (0.98)
* How often do you search for information about healthcare system via your colleges, neighbours or other persons (v10) *1-very unlikely to 4-very likely;*	2.26 (1.14)
*Suppose you search online for information about healthcare in the Netherlands, how likely would you be to consult the website of your local GGD (v11) *1-very unlikely to 4-very likely*	2.84 (1.06)
*Suppose you search online for information about healthcare in the Netherlands, how likely would you be to consult the online zorgkaart? (v12) *1-very unlikely to 4-very likely*	2.84 (1.23)
*Suppose you search online for information about healthcare in the Netherlands, how likely would you be to consult the webpage from your health insurer (v13) *1-very unlikely to 4-very likely*	3.03 (0.96)
*Suppose you search online for information about healthcare in the Netherlands, how likely would you be to consult social media such as Facebook or Twitter (v14) *1-very unlikely to 4-very likely*	1.81 (1.09)
**In the past 12 months, how often have you visited your family doctor for medical problems of yourself or of a family member? (v19) *1- never to 6 > 6 times;*	2.81 (1.57)
***Have you ever participated in breast cancer screening? (v59) 1 – yes, 0-no	0.28 (0.45)
***Have you ever participated in colon cancer screening? (v60) 1-yes; 0-no	0.32 (0.47)
***Have you ever participated in cervical cancer screening? (v61)1-yes; 0=no	0.42 (0.5)
****How confident are you when filling out medical forms? (v64) *1- non confident at all – 4-absolutely confident*	3.21 (0.86)
Variable name	Mean/median (SD)
****How often do you have trouble learning about medical conditions because you have trouble understanding written information? *1-never to 5-very often*	2.61 (1.53)
****How often do you let someone help you read hospital and doctor's materials? *1-never to 5 – very often*	2.20 (1.55)

* for variables v8 to v14 category coded 5 – “I do not know” is recoded as missing.

**for variable v19 categories 7- “I do not know” & 8 – “I will not say” are coded as missing.

*** for variables v59 to v61 categories 4- “I do not know” & 5 - “I will not say “are coded as missing.

****for variables v64-v66 categories 6 – “this do not apply to me is coded as missing.

In Table 2 we present results on the percentage of correct answers related to cancer knowledge between migrants (first- and second-generation) and non-migrants. In Table 3, we present results related to ATT – difference in knowledge between migrants and non-migrants for the three types of screening procedures. Results from the logit regression on matched samples are presented in Table 4, Table 5.

**Table 2:** Percentage of correct answers for three outcome variables among first-generation migrants, second-generation migrants, and non-migrants.

	Knowledge about breast cancer screening	Knowledge about colon cancer screening	Knowledge about cervical cancer screening
First generation non -Western migrants (*N* = 159)	27.7 % (*n* = 44)	17 % (*n* = 27)	24.5 % (*n* = 39)
Second generation non -Western migrants (*N* = 141)	28.4 % (*n* = 40)	14.2% (*n* = 20)	36.2 % (*n* = 51)
Non – migrants (*N* = 310)	44.5 % (*n* = 138)	29 % (*n* = 90)	31.6 %(*n* = 98)

**Table 3:** Differences in knowledge about breast cancer screening, colon cancer screening and cervical cancer screening among first- and second-generation migrants and non-migrants.

	ATT (knowledge about breast cancer screening)			ATT (knowledge about colorectal cancer screening)			ATT (knowledge about cervical cancer screening)		
	Treated	Controls	Difference	Treated	Controls	Difference	Treated	Controls	Difference
First non-Western generation migrants vs non-migrants	0.27	0.41	−0.14* (*p* < 0.01)	0.17	0.29	−0.12* (*p* < 0.01)	0.23	0.34	−0.11** (*p* < 0.08)
Second generation non-Western migrants vs non-migrants	0.28	0.44	−0.16* (*p* < 0.01)	0.14	0.27	**−**0.13* (*p* = 0.05)	0.36	0.35	0.01 (*p* = 0.916)

**Table 4:** Logistic regression for all three outcome variables on matched samples – subsample first generation migrants and non-migrants.

	Knowledge about breast cancer – *n* = 154				Knowledge about colorectal cancer – *n* = 224				Knowledge about cervical cancer – *n* = 166			
	*Beta coefficient*	*Z score*	*Odds ratio*	*P value*	*Beta coefficient*	*Z score*	*Odds ratio*	*P value*	*Beta coefficient*	*Z score*	*Odds ratio*	*P value*
Searching information about healthcare system via friends (V8)	−0.14	−0.54	0.87	0.589	0.26	1.17	1.11	0.242	−0.21	−0.76	0.81	0.450
Searching information via family (V9)	−0.02	−0.09	0.97	0.927	0.14	0.65	1.15	0.566	0.96*	3.33	2.59	**0.001**
Searching information about healthcare via webpage of formal public health institutions (V11)												
Searching information about healthcare system via zorgkart webpage (V12)	−0.82*	−2.98	0.44	0.003	0.21	0.97	1.17	0.333	0.09	0.38	1.10	0.702
Searching information about healthcare system through health insurance webpage (V13)	0.48**	1.76	1.62	0.07	−0.06	−0.28	0.97	0.781	−0.55*	−1.99	0.57	**0.04**
Searching information about healthcare system through Facebook or X (V14)	−0.26	−0.94	0.77	0.347	−0.36	−1.44	0.73	0.151	−0.32	−1.11	0.72	0.267
How often did you visit GP in last 12 months (V19)	0.35*	2.07	1.42	0.04	0.27*	2.10	1.27	0.035	0.01	0.08	1.01	0.936
Have you ever participated in breast cancer screening (V59)	−0.42	−0.98	0.65	0.326								
Have you ever participated in colon cancer screening (V60)					1.03*	3.07	2.08	0.002				
Have you ever participated in cervical cancer screening (V61)									1.20*	2.92	3.31	**0.003**
How confident are you when filling out medical forms? (V64)	0.30	1.12	1.35	0.263	−0.19	−0.88	0.99	0.381	−0.58**	−1.94	0.56	**0.05**
	Knowledge about breast cancer				Knowledge about colorectal cancer				Knowledge about cervical cancer			
	*Beta coefficient*	*Z score*	*Odds ratio*	*P value*	*Beta coefficient*	*Z score*	*Odds ratio*	*P value*	*Beta coefficient*	*Z score*	*Odds ratio*	*P value*
How often do you have trouble learning about medical conditions because you have trouble understanding written information? (V65)	0.12	0.48	1.12	0.630	0.10	0.58	1.08	0.560	0.13	0.48	1.14	0.631
How often do you let someone help you read hospital and doctor's materials? (V66)	−0.16	−0.67	0.85	0.505	−0.24	−1.28	1.12	0.202	−0.42	0.28	0.65	0.136
t	−0.09	−0.23	0.91	0.820	−0.41	−1.17	0.62	0.240	−1.03*	−2.50	0.35	**0.01**
Cons	−0.85	−0.65	0.42	0.514	−2.07*	−1.80	0.07	0.07	1.10	0.82	3.03	0.414
Pseudo R^2^	0.11				0.10			0.17				
Log likelihood	−89.22				−116.75			−91.74

**Table 5:** Logit regression for all three outcome variables on matched samples – subsample second generation migrants and non-migrants.

	Knowledge about breast cancer				Knowledge about colorectal cancer				Knowledge about cervical cancer			
	*Beta coefficient*	*Z score*	*Odds ratio*	*P value*	*Beta coefficient*	*Z score*	*Odds ratio*	*P value*	*Beta coefficient*	*Z score*	*Odds ratio*	*P value*
Searching information about healthcare system via friends (V8)	0.22	0.84	1.24	0.400	0.74*	2.31	2.10	0.02	−0.19	−0.76	0.82	0.448
Searching information via family (V9)	−0.14	−0.52	0.87	0.606	−0.74*	−2.36	0.47	0.01	0.27	1.05	1.30	0.295
Searching information about healthcare via webpage of formal public health institutions (V11)											1.29	0.263
Searching information about healthcare system via zorgkart webpage (V12)	−0.37	−1.53	0.69	0.125	0.08	0.33	1.09	0.740	0.26	1.12	0.86	0.534
Searching information about healthcare system through health insurance webpage (V13)	0.50*	2.15	1.65	0.03	0.13	0.56	1.14	0.576	−0.15	−0.62	0.75	0.275
Searching information about healthcare system through Facebook or X (V14)	0.03	0.10	1.02	0.921	−0.14	−0.47	0.86	0.639	−0.29	−1.09	1.38	0.04
How often did you visit GP in last 12 months (V19)	0.04	−0.26	0.95	0.794	**−**0.13	−0.75	0.87	0.456	0.31	2.06		
Have you ever participated in breast cancer screening (V59)	1.46*	2.18	4.31	0.03								
	Knowledge about breast cancer – *n* = 159				Knowledge about colorectal cancer – *n* = 179				Knowledge about cervical cancer – *n* = 164			
	*Beta coefficient*	*Z score*	*Odds ratio*	*P value*	*Beta coefficient*	*Z score*	*Odds ratio*	*P value*	*Beta coefficient*	*Z score*	*Odds ratio*	*P value*
Have you ever participated in colorectal cancer screening (V60)					0.71	1.13	2.05	0.260				
Have you ever participated in cervical cancer screening (V61)									2.12*	4.92	8.04	0.00
How confident are you when filling out medical forms? (V64)	−0.07	−0.29	0.92	0.770	0.55**	1.81	1.74	0.07	0.14	0.63	1.15	0.531
How often do you have trouble learning about medical conditions because you have trouble understanding written information? (V65)	0.32	1.14	1.38	0.253	−0.09	0.29	0.91	0.774	0.03	0.10	1.02	0.919
How often do you let someone help you read hospital and doctor's materials? (V66)	−0.58*	−2.07	0.55	0.04	−0.20	−0.55	0.82	0.584	−0.18	−0.72	0.83	0.471
t	−1.08*	−2.89	0.34	0.00	−1.48*	−3.20	0.22	0.001	−0.32	−0.85	0.72	**0.393**
Cons	0.06	0.05	1.06	0.963	−1.97	−1.24	0.14	0.215	−1.82	−1.40	0.16	0.162
Pseudo R^2^	0.14				0.16				0.19			
Log likelihood	−93.41				−80.21				−91.24			

Additionally, to answer our second question – which factors contribute to the differences between migrants and non-migrants, we have performed a Blinder -Oaxaca decomposition approach (B-O)(Oaxaca, 1973). This approach is usually used to explain which factors (personal characteristics, networks etc.) drive the differences between two groups. In our case, the Blinder-Oaxaca decomposition will divide differences between migrants and non-migrants into the part that is explained by other covariates (such as place where persons search for health information) and residuals – part of the difference that cannot be explained by observed covariances. Residuals, or “unexplained” parts, are very often used as a measure of discrimination, but they also show the effects of unobserved covariates. In other words, we will explain how much of the differences between migrants and non-migrants can be explained by the effect of covariances (so called covariate effects/explained components) and how much of the differences is not explained by covariates – unexplained effect. Although this technique was originally used to examine discrimination in the labour market, it is now also being used to measure health inequalities. The results are presented in Table 6.

**Table 6:** Blinder - Oaxaca decomposition: difference in knowledge about preventive breast cancer screening and cervical cancer screening between migrants and non-migrants.

	Dependent variable: breast cancer knowledge		Dependent variable: cervical cancer knowledge	
	B coefficient (SE)	P value	B coefficient (SE)	P value
First-generation migrants	0.44 (0.03)	0.000	0.33 (0.03)	0.000
Non-Migrants	0.27 (0.04)	0.000	0.23 (0.03)	0.000
difference	0.17 (0.05)	0.000	0.10 (0.05)	0.003
Explained	0.02 (0.02)	0.364	−0.01 (0.02)	0.973
Unexplained	0.15 (0.05)	0.002	0.09 (0.05)	0.004

## Results

Table 1 presents descriptive statistics for all variables included in this study. Here we can see that the average age in our sample is 47.25, while men and women are equally represented in the sample. The average net income is in accordance with Dutch national average (CBS, 2021). Also, we see the average answers for questions regarding the use of network for searching health information is from 2.26 to 3.04 for 5-items scale. A similar trend is observed for the use of online platforms in searching for health information – from 2.84 to 3.04.

Results from Table 2 show that the percentage of non-migrants who gave correct answers is higher than for first- and second-generation migrants for breast cancer screening and for colon cancer screening. Second-generation migrants do report more often correct answers (36,2 %) than non-migrants (31.6 %) for cervical cancer screening. The difference between first- and second-generation of migrants is lowest for knowledge about breast cancer screening *(*27.7% vs 28.4 %)

Table 3 shows a significant difference between first-generation migrants and non-migrants regarding all three outcome variables. This means that first-generation migrants do give a correct answer with a lower probability than non-migrants. The difference is largest for knowledge of breast cancer screening, while the smallest difference is observed for cervical cancer screening. On the other hand, second-generation migrants do not differ in their knowledge from non-migrants regarding their knowledge about cervical cancer screening. However, for second-generation migrants, significant differences are observed for knowledge about breast cancer screening (−0.16*) and colorectal cancer screening (−0.13*). Results from balancing tests show that PSM is the adequate analysis since selection bias imposed by covariates is reduced after the matching (see Appendix 1).

Table 4 presents the results from three logistic regressions on the matched samples. Here, we present the factors that can contribute to the probability of giving correct answers to the knowledge screening questions for all three types of cancer among the best-matched first-generation migrants and non-migrants. Our results show that respondents who use online health insurance web pages have a higher probability (62 % increase) of giving correct answers to breast knowledge question (irrespectively of their migration status). Respondents who use online sources such as zorgkaart web-pages do have a lower probability of giving a correct answer regarding breast cancer. Previous screening experiences also seems to matter, however, the effect differs by screening type. Respondents who already had a colorectal cancer screening have a higher probability to give the correct answer regarding the knowledge question about the eligible age for colorectal cancer screening. However, results are quite different for the cervical cancer screening. Here we see that respondents who use family members as source of information do give the correct answer with a higher probability. Also, respondents who use web-pages from health insurance companies as information sources have a higher probability of giving a non-correct answer. Additionally, we have performed standard logistic regression on the non-matched sample as a robustness check. As our results showfor first-generation migrants, when knowledge related to breast cancer screening is used as a dependent variable, migrant status is significant, but also socio-demographic variables such as gender, education and income are also significant. Hence, with simple logistic regression models we cannot say that differences in system knowledge are only related to migration status. For two other dependent variables, knowledge about colorectal cancer screening and cervical cancer screening, the migrant status was not significant. These results are different than when we use PSM (see Supplementary file, Table 1, Table 2, Table 3, Table 4, Table 5, Table 6) and show how the use of standard regressions can mask potentional differences.

The channels through which information about healthcare is searched for also play a role for the system knowledge in the subsample of second-generation migrants and non-migrants (Table 5). While seeking information via friends increases knowledge about colorectal cancer screening programmes, seeking information via family members has a negative effect. The use of online information tools is only relevant for breast cancer knowledge, but not for colorectal or cervical cancer knowledge. Our results from standard logistic regression are slightly different - migrant status is significant only when knowledge related to breast cancer screening is used as a dependent variable, again showing the implications of selection bias.

Table 6 shows the results of the Blinder-Oaxaca decomposition to examine if the ethnic gap between migrants and non-migrants can be explained by the covariates presented above. We hereby focus only on the groups where significant differences were found and sufficent observations were available for the decomposition analysis: this refers to first-generation migrants and knowledge about breast and cervical cancer (see Table 3). We observe that for first-generation migrants, the gap in knowledge regarding breast cancer screening and cervical cancer tests exist and it is significant (difference between two B coefficients is 0.17 and 0.10 respectively). Hence, people with a migration background are less knowledgeable about the eligibility age for breast and cervical cancer preventive screenings than people without a migration background. The gaps in system knowledge cannot be explained by covariates examined in this study – such as whether migrants use their network or web-pages from formal information providers (for example, health insurances) to seek information. Rather, this ethnic knowledge gap can be explained by non-observed covariates. Examples could include structural factors (system factors) such as variation in access to the education system or the way how healthcare systems provide access to information for migrants, but also cultural differences in health-related behaviour, or discrimination.

To conclude, factors such as using social networks for getting healthcare information or using Facebook can be associated with system knowledge (regression tables on matched samples) but they do not explain the gap in knowledge that exist between first-generation non-Western migrants and non-migrants.

## Discussion

In this paper we have examined the differences in system knowledge regarding cancer screenings between non-Western migrants (first and second generation) and non-migrants in the Netherlands. System knowledge is a complex construct as it covers aspects of organization, eligibility, and access to welfare domains such as healthcare. In this paper we focus on people's knowledge about the age eligibility criteria to participate in the following three different screening tests: breast, colorectal, and cervical cancer screening tests. While previous studies, mostly of qualitative nature, have examined system knowledge as a crucial factor contributing to underutilization of healthcare, we apply a quantitative approach to examine ethnic differences in system knowledge and potential explanatory factors of this ethnic gap.

Our results show that, after controlling for observable factors, people with a migration background indeed know significantly less about the age eligibility to access cancer preventive screening programs than people without a migration background. This is a crucial finding as previous qualitative studies have identified a lack of knowledge as a key barrier to access cancer screening programs (Gong et al., 2022; Hamidui et al., 2022). Hence, ethnic differences in knowledge gaps can contribute to the already existing ethnic gap in cancer preventive screening programs. However, we also observe a difference between first- and second-generation migrants. For knowledge about cervical cancer screening programs, second generation migrants are equally well informed about the age eligibility criteria than people without a migration background. A potential explanation for this finding could be that information about the HPV virus (which causes cervical cancer) is already disseminated via schools whom second generation migrants attend to the same extent as non-migrants.

A Blinder-Oaxaca decomposition also reveals that "traditional" factors identified in previous literature, such as socio-demographic characteristics (age, gender, income, education) or how and where people search for information related to the health system, do not explain variation in system knowledge between migrants and non-migrants. However, some of these factors do explain variation of system knowledge *within* groups. For example, people using the official insurance websites to search for information possess more knowledge about breast cancer prevention screening programs than people who do not use these formal information channels, irrespectively of their origin. Knowledge about cervical screening programs, on the other hand, is negatively associated with the use of insurance websites. Previous screening participation contributes to more system knowledge only in the case of colorectal cancer and not breast or cervical cancer. This suggests that for breast and cervical cancer knowledge of the system may influence uptake, but previous uptake does not necessarily influence system knowledge. This is important because lack of system knowledge can lead to lower uptake even among people who have been screened once already. Last, but not least we find that none of these factors explain ethnic differences in system knowledge. This suggests that the ethnic gap in system knowledge might relate to structural factors not examined in this study. Examples include the social system, education system, health system, but also culture. Also, institutional discrimination could be responsible for the ethnic gap in system knowledge as observed in other preventive services (Ante-Testard et al., 2021; Teshale and Tesema, 2023). This also means that the way in which migrants navigate the host country's education, housing and health systems, or even their social engagement, may contribute to differences in system knowledge related to all three screening tests.

**Limitations:** In this study we have used a one item scale to operationalize each of our dependent variables. The use of a multi-item scale can lead to more detailed results. As covariates in our regression models we used variables related to previous experience with screening tests. This can lead to potential endogeneity problems. Also, we have used survey data to examine the factors that can explain differences in system knowledge between migrants and non-migrants.A mixed method design that will combine results from survey data with qualitative interviews can lead to better insight into those factors.

In our opinion, **future research** should focus on a better understanding and operationalization of system knowledge within the preventive healthcare system. A preventive healthcare system is often seen as a tool to not only increase life expectancy among vulnerable groups, but also to decrease financial burden. Our results show that even people who have participated in cancer prevention screening programs lack knowledge about the eligibility criteria of this program. Furthermore, we applied a PMS procedure to control for selection biases. However, the use of longitudinal data can lead to a better estimation of causal relations between explanatory factors and system knowledge and the development of ethnic differences in system knowledge over time.

**Policy recommendation:** Following our results, we suggest policy makers to use a transdisciplinary approach to disseminate information related to system knowledge. This should include cooperation and co-participation with people with a migration background, insurance companies and researchers. For example, in the case of mammography, information in the Netherlands is available in 6 different languages online. However, the first invitation letter for screening is sent only in Dutch. Taking into account that women with migration background possess lower levels of Dutch language skills, they have to depend on their relatives to read the letter. Furthermore, the use of digital health services is lower the older people get, particularly among the migrant population. Since breast cancer and colorectal cancer screening programs are targeted at people above the age of 50, policy makers should consider the cooperation with various stakeholders, including health centers, GP, but also migrant groups, to overcome those structural barriers.

## CRediT authorship contribution statement

**Dr Jelena Arsenijevic:** Writing – review & editing, Writing – original draft, Methodology, Formal analysis. **Dr Verena Seibel:** Writing – review & editing, Writing – original draft.

## Declaration of competing interest

The authors declare that they have no known competing financial interests or personal relationships that could have appeared to influence the work reported in this paper.

## Appendix Supplementary materials

Image, application 1

## References

[bib0001] Abeje G., Azage M. (2015). Hepatitis B vaccine knowledge and vaccination status among health care workers of Bahir Dar City administration, Northwest Ethiopia: a cross sectional study. BMC Infect. Dis..

[bib0002] Adunlin G., Cyrus J.W., Asare M., Sabik L.M. (2019). Barriers and facilitators to breast and cervical cancer screening among immigrants in the United States. J. Immigr. Minor. Health.

[bib0003] Ante-Testard P.A., Temime L., Jean K. (2021).

[bib0004] Arsenijevic J., Tummers L., Bosma N. (2020). Adherence to electronic health tools among vulnerable groups: systematic literature review and meta-analysis. J. Med. Internet Res..

[bib0005] Arsenijevic J., Groot W. (2017). Advocated but Sidelined: health promotion for the elderly in the Netherlands. Public Health Management/Zdrowie Publiczne i Zarzadzanie.

[bib0006] Austin K., Power E., Solarin I., Atkin W., Wardle J., Robb K. (2009). Perceived barriers to flexible sigmoidoscopy screening for colorectal cancer among UK ethnic minority groups: a qualitative study. J. Med. Screen..

[bib0007] Bell S., Edelstein M., Zatoński M., Ramsay M., Mounier-Jack S. (2019). ‘I don't think anybody explained to me how it works’: qualitative study exploring vaccination and primary health service access and uptake amongst Polish and Romanian communities in England. BMJ Open.

[bib0008] Berens E.M., Yilmaz-Aslan Y., Spallek J., Razum O. (2016). Determinants of mammography screening participation among Turkish immigrant women in Germany–a qualitative study reflecting key informants' and women's perspectives. Eur. J. Cancer Care (Engl).

[bib0009] Borjas G.J., Kauppinen I., Poutvaara P. (2019). Self-selection of emigrants: theory and evidence on stochastic dominance in observable and unobservable characteristics. Econ. J..

[bib0010] Bronzwaer M.E., Depla A.C., Van Lelyveld N., Spanier B.W., Oosterhout Y.H., van Leerdam M.E., Spaander M.C., Dekker E., van Haastert M., Keller J. (2019). Quality assurance of colonoscopy within the Dutch national colorectal cancer screening program. Gastrointest. Endosc..

[bib0011] Buskwofie A., David-West G., Clare C.A. (2020). A review of cervical cancer: incidence and disparities. J. Natl. Med. Assoc..

[bib60] Centraal Bureau voor de Statistiek. (2021). Retrieved February, 2023 from: https://opendata.cbs.nl/statline/#/CBS/nl/dataset/84494NED/table?ts=1722511079382.

[bib0012] de Hoon M., Vink M., Schmeets H. (2021). On the move again? residential trajectories of refugees after obtaining asylum in the Netherlands. Popul. Space Place.

[bib0013] de Jong P.W., de Valk H.A. (2023). Emigration of the Western European second generation: is having immigrant parents a predictor of international migration?. J. Ethn. Migr. Stud..

[bib0015] Dsouza J.P., Broucke S.V.d., Pattanshetty S., Dhoore W. (2022). A comparison of behavioural models explaining cervical cancer screening uptake. BMC Womens Health.

[bib0017] Ferdous M., Lee S., Goopy S., Yang H., Rumana N., Abedin T., Turin T.C. (2018). Barriers to cervical cancer screening faced by immigrant women in Canada: a systematic scoping review. BMC Womens Health.

[bib0018] Ferlay J., Soerjomataram I., Dikshit R., Eser S., Mathers C., Rebelo M., Parkin D.M., Forman D., Bray F. (2015). Cancer incidence and mortality worldwide: sources, methods and major patterns in GLOBOCAN 2012. Int. J. Cancer.

[bib0019] Fracheboud J., De Koning H., Boer R., Groenewoud J., Verbeek A., Broeders M., Van Ineveld B., Hendriks J., De Bruyn A., Holland R. (2001). Nationwide breast cancer screening programme fully implemented in The Netherlands. The Breast.

[bib0020] Gardner D.G., Cummings L.L., Dunham R.B., Pierce J.L. (1998). Single-item versus multiple-item measurement scales: an empirical comparison. Educ. Psychol. Meas..

[bib0021] Gmeinder, M., Morgan, D., & Mueller, M. (2017). How much do OECD countries spend on prevention?.

[bib0022] Gong J., Kampadellis G., Kong Q., Spijker W. (2022). Factors determining non-attendance in breast cancer screening among women in the Netherlands: a national study. Health Promot. Int..

[bib0023] Hamdiui N., Marchena E., Stein M.L., van Steenbergen J.E., Crutzen R., van Keulen H.M., Reis R., van den Muijsenbergh M.E., Timen A. (2022). Decision-making, barriers, and facilitators regarding cervical cancer screening participation among Turkish and Moroccan women in the Netherlands: a focus group study. Ethn Health.

[bib0024] Harding C., Seal A., Duncan G., Gilmour A. (2017). General practitioner and registrar involvement in refugee health: exploring needs and perceptions. Aust. Health Rev..

[bib0025] Hartman E., van den Muijsenbergh M.E., Haneveld R.W. (2009). Breast cancer screening participation among Turks and Moroccans in the Netherlands: exploring reasons for nonattendance. Eur. J. Cancer Prev..

[bib0026] Hertzum-Larsen R., Kjær S.K., Frederiksen K., Thomsen L.T. (2019). Participation in cervical cancer screening among immigrants and Danish-born women in Denmark. Prev. Med..

[bib0027] Indicators, O. (2021). Indicator overview: country dashboards and major trends.

[bib0028] Kennedy, S., McDonald, J.T., & Biddle, N. (2006). The healthy immigrant effect and immigrant selection: evidence from four countries.

[bib0030] Marques P., Nunes M., Antunes M.d.L., Heleno B., Dias S. (2020). Factors associated with cervical cancer screening participation among migrant women in Europe: a scoping review. Int. J. Equity Health.

[bib0032] Mehrara L., Olaug Gjernes T.K., Young S. (2022). Immigrant women's experiences with Norwegian maternal health services: implications for policy and practice. Int. J. Qual. Stud. Health Well-being.

[bib0033] Norredam M., Agyemang C., Hoejbjerg Hansen O.K., Petersen J.H., Byberg S., Krasnik A., Kunst A.E. (2014). Duration of residence and disease occurrence among refugees and family reunited immigrants: test of the ‘healthy migrant effect'hypothesis. Trop. Med. Int. Health.

[bib0034] Oaxaca R. (1973). Male-female wage differentials in urban labor markets. Int. Econ. Rev. (Philadelphia).

[bib0035] Özkan İ., Taylan S. (2021). Barriers to women's breast cancer screening behaviors in several countries: a meta-synthesis study. Health Care Women Int.

[bib0037] Patel G., Brosnan C., Taylor A. (2023). Understanding the role of context in health policy implementation: a qualitative study of factors influencing traditional medicine integration in the Indian public healthcare system. Health Sociology Review.

[bib0038] Rosenstock I.M., Strecher V.J., Becker M.H. (1988). Social learning theory and the health belief model. Health Educ. Q..

[bib0040] Ruijer E., Arsenijevic J. (2020). Global Equity in Administration.

[bib0041] Salad J., Verdonk P., de Boer F., Abma T.A. (2015). A Somali girl is Muslim and does not have premarital sex. is vaccination really necessary?” a qualitative study into the perceptions of Somali women in the Netherlands about the prevention of cervical cancer. Int. J. Equity Health.

[bib0042] Scherpenzeel A. (2011). Data collection in a probability-based internet panel: how the LISS panel was built and how it can be used. Bulletin of Sociological Methodology/Bulletin de Méthodologie Sociologique.

[bib0043] Seibel V. (2019). Determinants of migrants’ knowledge about their healthcare rights. Health Sociol. Rev..

[bib0044] Seibel V. (2023). The impact of migrants' knowledge about their social rights on their subjective wellbeing. Front. Polit. Sci..

[bib0045] Seibel V. (2021). What do migrants know about their childcare rights? A first exploration in West Germany. J. Int. Migr. Integr..

[bib0046] Seibel V., Renema J.A. (2021). Migrants’ and natives’ attitudes toward public healthcare provision in Denmark, Germany, and the Netherlands. Int. J. Public Opin Res..

[bib0047] Sinning M., Hahn M., Bauer T.K. (2008). The Blinder–Oaxaca decomposition for nonlinear regression models. Stata J..

[bib0048] Straiton M.L., Myhre S. (2017). Learning to navigate the healthcare system in a new country: a qualitative study. Scand J. Prim. Health Care.

[bib0049] Sung H., Ferlay J., Siegel R.L., Laversanne M., Soerjomataram I., Jemal A., Bray F. (2021). Global cancer statistics 2020: GLOBOCAN estimates of incidence and mortality worldwide for 36 cancers in 185 countries. CA Cancer J. Clin..

[bib0050] Suphanchaimat R., Kantamaturapoj K., Putthasri W., Prakongsai P. (2015). Challenges in the provision of healthcare services for migrants: a systematic review through providers’ lens. BMC Health Serv. Res..

[bib0051] Teshale A.B., Tesema G.A. (2023).

[bib0052] Van Hemelrijck W.M.J., Suggs L.S., Grossi A.A., Schröder-Bäck P., Czabanowska K. (2019). Breast cancer screening and migrants: exploring targeted messages for Moroccan migrant women in Brussels. Ethn. Health.

[bib0054] Vu M., Yu J., Awolude O.A., Chuang L. (2018). Cervical cancer worldwide. Curr. Probl. Cancer.

[bib0055] Wake A.D. (2020). Knowledge, attitude, practice, and associated factors regarding the novel coronavirus disease 2019 (COVID-19) pandemic. Infect. Drug Resist..

[bib0056] WHO (2024). https://www.who.int/news/item/01-02-2024-global-cancer-burden-growing–amidst-mounting-need-for-services.

[bib0057] Willis K., Collyer F., Lewis S., Gabe J., Flaherty I., Calnan M. (2016). Knowledge matters: producing and using knowledge to navigate healthcare systems. Health Sociol. Rev..

[bib0058] Woudstra A.J., Dekker E., Essink-Bot M.L., Suurmond J. (2016). Knowledge, attitudes and beliefs regarding colorectal cancer screening among ethnic minority groups in the N etherlands–a qualitative study. Health Expect..

[bib0059] Xiang V., Parackal S., Gurung G., Subramaniam R.M. (2023). Asian migrants navigating New Zealand primary care: a qualitative study. J. Prim. Health Care.

