# Supplementary material for: Do immigrants know less than natives about cancer screening tests? – the case of Netherlands
Source: J Migr Health. 2024 Jul 29;10:100258. doi: 10.1016/j.jmh.2024.100258 (PMC11363821; doi:10.1016/j.jmh.2024.100258)
Supplement: Supplementary file 1 [file mmc1.docx]

Table 1: Logistic regression without matching – dependent variable knowledge about breast cancer screening, main independent variable: first generation migrants

Table 2: Logistic regression without matching – dependent variable knowledge about cervical cancer screening, main independent variable: first generation migrants

Table 3: Logistic regression without matching – dependent variable knowledge about colorectal cancer screening, main independent variable: first generation migrants

Table 4: Logistic regression without matching – dependent variable knowledge about breast cancer screening, main independent variable: second generation migrants

Table 5: Logistic regression without matching – dependent variable knowledge about cervical cancer screening, main independent variable: second generation migrants

Table 6: Logistic regression without matching – dependent variable knowledge about colorectal cancer screening, main independent variable: second generation migrants

Table 7: Balancing test for matching first generation migrants and non-migrants, dependent variable: knowledge about breast cancer screening

Table 8: Balancing test for matching first generation migrants and non-migrants, dependent variable: knowledge about cervical cancer screening

Table 9: Balancing test for matching first generation migrants and non-migrants, dependent variable: knowledge about colorectal cancer screening
